# Cytokines and chemokines modulate the growth of pituitary adenoma/neuroendocrine tumors: preliminary results of a monocenter prospective pilot study

**DOI:** 10.1007/s11102-025-01505-4

**Published:** 2025-03-10

**Authors:** Sabrina Chiloiro, Pier Paolo Mattogno, Flavia Angelini, Antonella Giampietro, Alessandra Vicari, Greis Konini, Federico Valeri, Amato Infante, Natalia Cappoli, Rosalinda Calandrelli, Liverana Lauretti, Simona Gaudino, Marco Gessi, Guido Rindi, Alessandro Olivi, Laura De Marinis, Antonio Bianchi, Francesco Doglietto, Alfredo Pontecorvi

**Affiliations:** 1 Facoltà di Medicina e Chirurgia, Università Cattolica del Sacro Cuore, Rome, Italy; 2 Dipartimento di Endocrinologia, Diabetologia e Medicina Interna, Fondazione Policlinico Universitario A. Gemelli, Istituto di Ricovero e cura a carattere scientifico (IRCCS), Rome, Italy; 3 Department of Neurosurgery, Fondazione Policlinico Universitario A. Gemelli, Istituto di Ricovero e cura a carattere scientifico (IRCCS), Rome, Italy; 4 Department of Radiological Sciences, Fondazione Policlinico Universitario A. Gemelli, Istituto di Ricovero e cura a carattere scientifico (IRCCS), Rome, Italy; 5 Neuropathology Unit, Fondazione Policlinico Universitario A. Gemelli, Istituto di Ricovero e cura a carattere scientifico (IRCCS), Rome, Italy

**Keywords:** Pituitary adenoma, PitNET, Microenvironment, Immune cells, Lymphocytes, Macrophages, Acromegaly

## Abstract

**Introduction:**

Cytokine and chemokines have been recognized to be involved in the progression and prognosis of pituitary adenoma/neuroendocrine tumors (PAs/PitNETs), also known as pituitary adenomas. We aim to investigate the expression of cytokine and chemokine in PAs/PitNETs, and their association with PAs/PitNETs clinical and biological behavior.

**Patients and methods:**

A prospective and monocenter study was performed on 16 patients diagnosed for PAs/PitNETs. Cytokine and chemokine were detected on freshly collected PAs/PitNETs samples. Tumor infiltering immune cells were investigated on formally fixed and paraffin-embedded PAs/PitNETs samples. Clinical, biochemical, molecular and morphological data were collected from patients’ medical records.

**Result:**

Out of 72 patients with PAs/PitNETs that underwent surgical removal at the Neurosurgery Division of our Institution between January and June 2023, sixteen patients were enrolled in the study. Out of 42 cytokines and chemokines that we investigated, we found that the expressions of the growth-regulated oncogene (GRO)/CXCL1, thymus- and activation-regulated chemokine (TARC)/CCL17 and epidermal growth factor (EGF) were higher in invasive tumors than in not-invasive ones (respectively *p* = 0.01, *p* = 0.002 and *p* = 0.002). The EGF expression was higher in tumors with a MIB1 > 3% than in those with MIB1 < 3% (*p* = 0.014). A positive correlation was detected between the expressions of EGF and CXCL1 (*p* = 0.003, r: 0.7), EGF and GRO-a (*p* = 0.01, r:0.61), and the number of tumors infiltering CD68 + macrophages and the expression of CCL2 (*p* = 0.008, *r* = 0.695).

**Conclusion:**

Our preliminary results support that in PAs/PitNETs, the cytokines and chemokines generate an immune network, that may contribute to regulating the cell proliferation and pattern of growth.

**Supplementary Information:**

The online version contains supplementary material available at 10.1007/s11102-025-01505-4.

## Introduction

Cytokines and chemokines are components of the tumor microenvironment (TME) in pituitary adenoma/neuroendocrine tumors (PAs/PitNETs), secreted by tumor cells (TCs), tumor infiltering-immune cells (TICs) and tumor-associated fibroblast [1–3].

Specific cytokines/chemokines, such as the C-X-C motif ligand (CXCL)-12, C-C motif (CCL)5, CCL17, interleukin (IL)-8, IL-6, IL-1, IL-2, IL-17, tumor necrosis factor (TNF)-alpha, and vascular endothelial growth factor (VEGF), modulate TME composition, hormone secretion, tumors proliferation and invasion [3–5]. Studies on soluble factors of TME suggested a potential role as treatment target [6], such as VEGF-inhibitor [7].

In this study, we aimed to investigate the cytokine/chemokine systems in PAs/PitNETs, and their association with clinical and biological behavior, such as hormone secretion, pathology subtype, proliferation, invasive growth and disease outcome.

## Patients and methods

A prospective, observational, monocenter study was performed on patients naïve to medical therapy and underwent surgery for PAs/PitNETs in 2023 and followed up at our institution for at least a year.

Inclusion and exclusion criteria were reported in supplementary section.

### Study protocol

The following data were collected: gender, age at diagnosis, hormone status, tumor morphology (tumor maximum diameter, tumor volume, tumor extension and invasive growth), disease outcome, according to specific disease guidelines for lactotroph, somatotroph, corticotroph tumors [8–11]. For the study experiments, the resected tumor was formally fixed and paraffin-embedded for pathological diagnosis and for the detection of TIC, as reported in our previous studies [12–14]. Moreover, a sample of PAs/PitNET was freshly collected and reserved for the simultaneous detection of 42 cytokines and chemokines (Human Cytokine Antibody Array ab133997, Abcam). The intensity score (IS) of each spot-on membrane was corrected for background intensity and normalized to positive control spots on reference array. In each assay, a negative control was included. A health control was tested (total tissue protein lysate of human adult pituitary gland Leinco), following the same procedures of the cases (supplementary Fig. 1). Anti-pituitary antibodies (APA) were detected on serum collected the day before the surgical removal of the PAs/PitNET, according to our clinical practice, though immunofluorescence [15]. Experimental procedures are detailed in supplementary section.

## Result

Out of 72 patients with PAs/PitNETs that underwent surgical removal at the Neurosurgery Division of our Institution between January and June 2023, sixteen patients were enrolled in the study. Clinical features of the study cohort are summarized in Table 1. IL-1b, IL-2, IL-3, IL-8, CCL2, EGF, CXCL1, CXCL1 -a, CCL5, SDF1, CCL17, MDC, VEGF, PDGF88 and the leptin were positively detected in samples of tumors: IL-2, IL-8, GRO, CCL5 and SDF-1 were detected in all samples. The other cytokines/chemokines were not identified in samples. No cytokines/chemokines were detected in control (supplementary Fig. 1).

**Table 1:** Clinical, demographic, morphology and pathology feature of the study population

	Gender	Age	Tumors subtype *	Trascription Factor	MIB-1	Endocrine syndrome	Tumor invasion	Cavernous sinus invasion	Outcome
Pt1	F	76	Somatotroph	Pit-1	< 1%	Acromegaly	No	Knosp 0–2	S.C.
Pt2	F	29	Lactotroph	Pit-1	2–3%	Hyperprolactinemia	No	Knosp 0–2	S.C.
Pt3	F	54	Somatotroph	Pit-1	1	Acromegaly	No	Knosp 0–2	S.C.
Pt4	M	43	Gonadotroph	SF1, GATA-3	1–2%	Not endocrine symptoms	No	Knosp 0–2	S.R.
Pt5	F	39	Somatotroph	Pit-1	< 1%	Acromegaly	Cavernous sinus	Knosp 3–4	Controlled with Pasireotide Lar
Pt6	F	63	Somatotroph	Pit-1	< 1%	Acromegaly	Cavernous sinus	Knosp 0–2	S.C.
Pt7	M	24	Somatotroph	Pit-1	1–2%	Acromegaly	Cavernous sinus	Knosp 0–2	S.C.
Pt8	M	84	Gonadotroph	SF1, GATA-3	< 1%	Not endocrine symptoms	Cavernous sinus	Knosp 3–4	S.R.
Pt9	F	76	Gonadotroph	SF1, GATA-3	1–2%	Not endocrine symptoms	No	Knosp 0–2	S.R.
Pt10	M	72	Gonadotroph	SF1, GATA-3	1–2%	Not endocrine symptoms	Sphenoidal sinus	Knosp 0–2	S.C.
Pt11	F	77	Gonadotroph	SF1, GATA-3	1–2%	Not endocrine symptoms	Sphenoidal sinus	Knosp 0–2	S.C.
Pt12	F	55	Corticotroph	TPIT	< 1%	Hypercortisolism	Sphenoidal sinus	Knosp 3–4	Steroidogenesis inhibitors
Pt13	M	59	Gonadotroph	SF1, GATA-3	1–2%	Not endocrine symptoms	Cavernous sinus	Knosp 3–4	S.R.
Pt14	F	29	Corticotroph	TPIT	5–7%	Hypercortisolism	Dura mater	Knosp 0–2	S.C.
Pt15	F	33	Corticotroph	TPIT	5–7%	Hypercortisolism	No	Knosp 0–2	S.C.
Pt16	M	68	Lactotroph	Pit-1	3–4%	Hyperprolactinemia	Cavernous sinus, sphenoidal sinus, clivus	Knosp 3–4	Controlled with Cabergoline

(*) Subtypes were classified according to 2022 WHO Classification of Endocrine organs. SC: surgical cure, SR: stable residual, Pit-1: pituitary-specific transcription factor, GATA-3: binding protein 3, T-PIT: T-box transcription factor, SF-1: steroidogenic factor-1

A positive correlation was detected between the intensity score of EGF and CXCL1 (*p* = 0.003, r:0.7), of EGF and CXCL1-a (*p* = 0.01, r:0.61); of EGF and CCL5 (*p* = 0.004, r:0.68); and of CXCL1 and CCL5 (*p* = 0.003, *r* = 0.684). An inverse correlation was identified between intensity score of CXCL1 and IL-2 (*p* = 0.002, r:-0.7). A positive correlation was detected between the number of tumor-infiltering CD68 + macrophages and the CCL2 intensity score (*p* = 0.008, *r* = 0.695). No correlation was identified between the presence of circulating anti-pituitary antibodies and presence of tumor cytokines and chemokines.

As reported in supplementary Table 1, no differences were identified in subtypes of pituitary tumors and the presence of cytokines and chemokines in tumor samples.

### Cytokines and chemokines as markers of tumor behavior

**Fig. 1:**
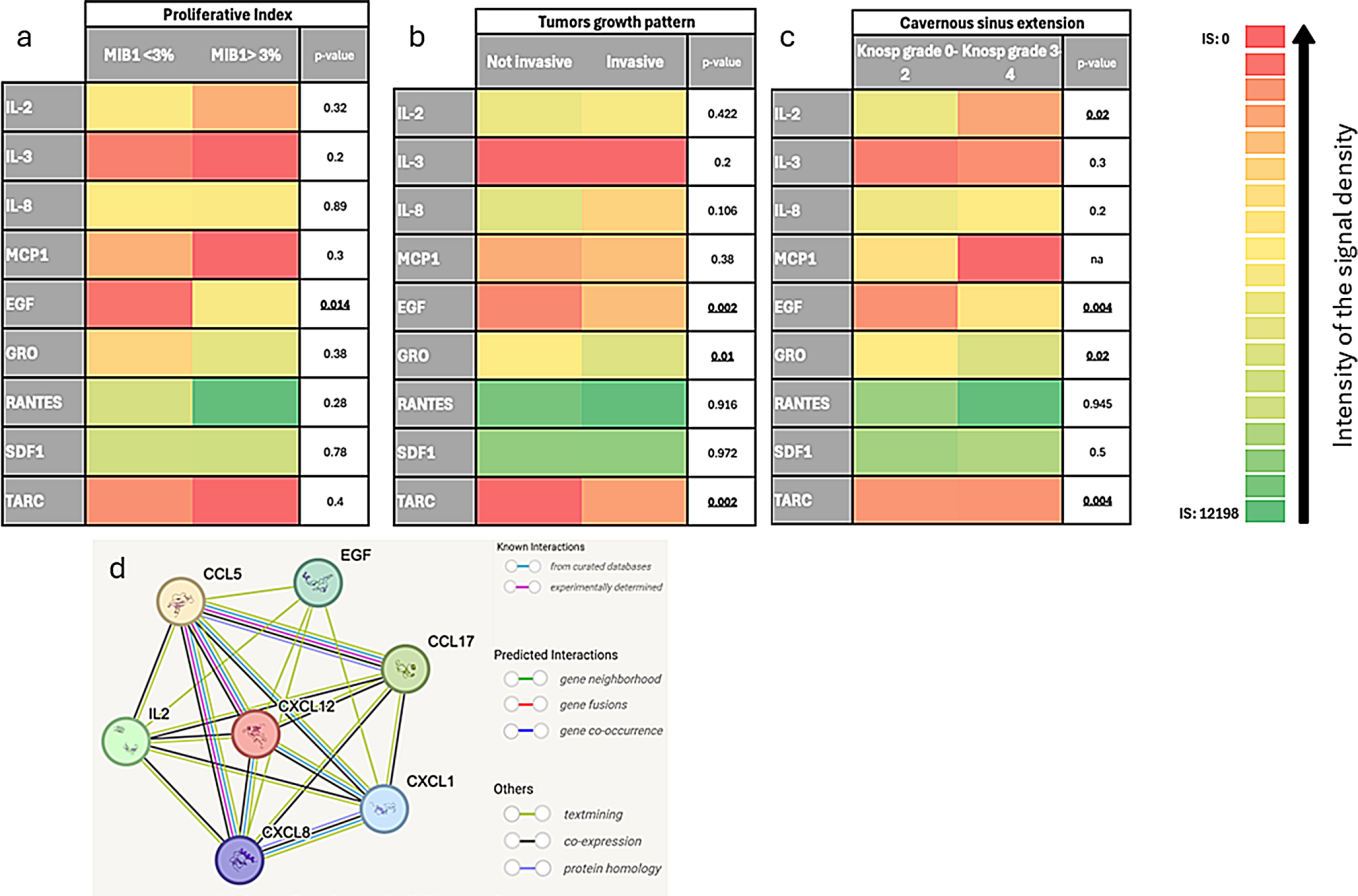
Intensity graph representing the correlation between the intensity of the signal density for each cytokine and chemokine and (**a**) the proliferative index (Mib1 < 3% or Mib > 3%), (**b**) tumor growth pattern (not invasive vs invasive), (**c**) cavernous sinus extension (Knosp’s grade 0–2 vs Knosp’s grade 3 and 4). Box d outlines molecular pathway of EGF, GRO (CXCL1), RANTES (CCL5), IL-2, IL-8 (CXCL8), TARC (CCL17), SDF-1 (CXCL12); the box d was generated from String version 12.0

EGF was significantly more expressed in tumors with MIB1 > 3% (IS:1515 IQR:1642), than in tumor with MIB1 *≤* 3% (IS: 81 SD: 292, *p* = 0.014); the IS of the other cytokines/chemokines did not differ in tumor with MIB1 *≤* 3% and in those with MIB1 > 3% (Fig. 1a).

Invasive PAs/PitNETs had a higher expression of EGF (IS:465 IQR:1232, *p* = 0.002), of CXCL1 (IS:1756 IQR:2055, *p* = 0.01), of CCL17 (IS:351 IQR:609, *p* = 0.002) then not invasive PAs/PitNETs (EGF IS:260, IQR:520; GRO:718 IQR:890; CCL17:147, IQR:443), as reported in Fig. 1b. PAs/PitNETs with invasion of the cavernous sinus expressed higher levels of EGF (IS:652 IQR:1457, *p* = 0.004), of CXCL1 (IS:2170 IQR:2308, *p* = 0.02) and lower IL-2 levels (IS:326 IQR:729, *p* = 0.02), then not invasive cavernous sinus PAs/PitNETs (EGF IS:212 IQR:476; CXCL1 IS: 719 IQR:859, IL-2 IS:326 IQR:729), Fig. 1c.

## Discussion

In this study, we proved that specific cytokines and chemokines, such as EGF, CXCL1, CCL17, CCL5, CXCL12, IL-2, IL-8, are expressed in PAs/PitNETs and act modulating pattern of growth and proliferation: higher expression of EGF, CXCL1, CCL17 and lower expression of IL-2 were identified in invasive PAs/PitNETs. Moreover, we found a positive correlation between the expressions of EGF, CXCL1, and CCL5, and between the expressions of CXCL1 and CCL5; and a negative correlation between the expression of CXCL1 and IL-2, generating an immune network (Fig. 1d).

These results are consistent with previous literature, as we found a significant increased expression of EGF in invasive and proliferative PAs/PitNETs. EGF regulates the expression of the LH, GH and PRL mRNA [16], the cell mitogenic activity in corticotroph cells [17] and in aggressive lactotroph PAs/PitNETs [18]. The EGF expression in PAs/PitNETs suggested also the opportunity of target therapy against EGF pathway in aggressive tumors [19]. In-vitro studies suggested that lapatinib (an EGFR-inhibitor) reduce corticotroph tumor cell proliferation [20]. Data on humans are limited to few patients with aggressive lactotroph PitNETs treated with EGFR-inhibitor, with variable outcomes [19, 21]. Moreover, we identified that the EGF expression positively correlated with the expression of CXCL1 and CCL5. In PAs/PitNETs, CXCL1 recruits macrophages and neutrophils, promoting tumor progression [22], and CCL5 enhances the tumor invasiveness [23].

In this cohort, we found a negative correlation between expression of CXCL1 and IL-2. The autocrine/paracrine action of IL-2 in promoting ACTH and PRL secretion, and proliferation of pituitary cells were widely reported [24, 25]. More recently, the IL-2 expression was reported also in secreting corticotroph tumors [26]. Interestingly in all PAs/PitNET samples that were analyzed in this study, CXCL12 and IL-8 were detected. The presence of IL-8 was reported mainly in somatotropinomas [27], with higher levels in more aggressive tumors [22]. At least, in this study, we found a positive correlation between the number of tumor infiltering-CD68 + macrophages and CCL2, a chemokine able to recruit monocytes/macrophages at the site of inflammation [28].

The main limitations of this study are due to the small sample size that can affect the statistical power of our results, the non-homogenous cohort of PAs/PitNETs, and the lack of validation of results though different experimental models, such as RNA and immunohistochemistry analysis, or primary cultures/cell lines of pituitary adenoma cells. Despite these limitations, the strength of this study was that our experiments were conducted on human tumor samples rather than on cell cultures, providing in-vivo data that reflect more realistically and comprehensively the dynamic interplay between tumor cells and cytokines/chemokines, and providing also preliminary data on their potential role in regulation tumor proliferation and invasion.

In conclusion, the preliminary results of this pilot study support that in human PAs/PitNETs, cytokines and chemokines generate an immune network, that may contribute to regulate tumor behavior. Additional studies on larger series may corroborate our results, possibly supporting the investigation of cytokines and chemokines system as target for the personalized therapies.

## Electronic supplementary material

Below is the link to the electronic supplementary material.

Supplementary Material 1

Supplementary Material 2

Supplementary Material 3

Supplementary Material 4

## Data Availability

The data sets generated during and/or analyzed during the current study are not publicly available, but are available from the corresponding author on reasonable request.
