# Supplementary material for: Cytokines and chemokines modulate the growth of pituitary adenoma/neuroendocrine tumors: preliminary results of a monocenter prospective pilot study
Source: Pituitary. 2025 Mar 10;28(2):37. doi: 10.1007/s11102-025-01505-4 (PMC11893686; doi:10.1007/s11102-025-01505-4)
Supplement: Supplementary file 1 — Supplementary Material 1 [file 11102_2025_1505_MOESM1_ESM.docx]

**Study protocol**

*Study population*

Patients were consecutively enrolled in the study according to the following inclusion/exclusion criteria.

Inclusion criteria:

(1) clinical or radiological diagnosis of PitNETs;

(2) first-line treatment with surgical removal performed between January 2023 and June 2023;

(3) histopathological diagnosis of PAs/PitNETs, according to 2022 WHO Classification (1) of tumors of Endocrine organs;

(4) at least one year follow-up at the Pituitary Unit;

(5) availability of freshly collected samples and of formalin-fixed and paraffin-embedded (FFPE) tissue for experimental analysis;

(6) availability of serum samples collected the day before the surgical removal of the pituitary tumor;

(7) agreement to participate to the study, by signing an informed consent.

Exclusion criteria were:

(1) neoadjuvant medical treatments;

(2) radiotherapy on head and neck within 10 years before pituitary surgery;

(3) sellar apoplexy.

*Pathology analysis*

Pathology analyses were conducted according to our clinical practice and as previous reported (2–4). All specimens were examined for the expression of pituitary hormones and transcription factors, pituitary-specific transcription factor (Pit-1), GATA binding protein 3 (GATA-3), T-box transcription factor (T-PIT), steroidogenic factor-1 (SF-1); proliferative index (MIB1); and TME components. The MIB1 index was expressed as percent of positive nuclei in “hot spot” area. The number of clusters of differentiation (CD)-3+, CD20+, CD138+, CD4+, CD8+ lymphocytes and of CD68+ macrophages was expressed as the total of positive cells in four high-power fields (1 HPF: 0.25 mm2). Fields were randomly selected within tumor tissue, avoiding areas close to vessels or in areas of hemorrhage. The positive cells of four sequential fields were counted. Cells were considered positive only if the cellular nucleus was identified. Antibodies applied were detailed in supplementary table 1.

| **Antibody (clone)** | **Supplier** |
| --- | --- |
| **KI67 clone MM1 RRID:** [**AB_442101**](https://scicrunch.org/resolver/%20AB_442101) | Leica Microsystems |
| **p53 clone D07 RRID:** [**AB_442120**](https://scicrunch.org/resolver/AB_442120) | Leica Microsystems |
| **CD4 clone 4B12 RRID:** [**AB_10554438**](https://scicrunch.org/resolver/AB_10554438) | Leica Microsystems |
| **CD8 clone 4B11 RRID:** [**AB_10554590**](https://scicrunch.org/resolver/AB_10554590) | Leica Microsystems |
| **CD68 clone 514H12 RRID:** [**AB_10554758**](https://scicrunch.org/resolver/AB_10554758) | Leica Microsystems |
| **GATA clone L50-823** | Roche diagnostics SpA |
| **T-PIT AMAB91409 clone CL6251** | Sigma-Aldrich |
| **SF-1 EPR19744 RRID: AB 217317** | ABCAM |
| **PIT1 clone nbp1-92273 RRID:** [**AB_11030310**](https://scicrunch.org/resolver/AB_11030310) | Novus Ultra View |

Supplementary table 1. Details of the primary antibodies used for immunohistochemistry.

*Cytokines and chemokines detection*

Human Cytokine Antibody Array (ab133997, Abcam) was used for the simultaneous detection of 42 cytokines in each sample according to the manufacturer’s instructions: epithelial neutrophil-activating protein 78 (ENA-78), granulocyte colony-stimulating factor (GCSF), granulocyte-macrophage colony-stimulating factor (GM-CSF), CXCL1/growth-regulated oncogene (GRO), alpha-GRO, CC Chemokine I-309 (I-309), alpha-IL-1, beta-IL1, IL-2, IL-3; IL-4; IL-5; IL-6; IL-7; IL-8; IL-10; IL-12p40/p70; IL-13; IL-15; gamma-interferon (INF); CCL2/monocyte chemoattractant protein (MCP)-1; MCP-2; MCP-3; macrophage colony-stimulating factor (MCSF); macrophage-derived chemokine (MDC), monokine induced by interferon-gamma (MIG); macrophage inflammatory protein 1 delta (delta-MIP-1); CCL5/regulated upon activation, normal T cell expressed and secreted (RANTES), stem cell factor (SCF), stromal cell-derived factor-1 (SDF-1); thymus- and activation-regulated chemokine (TARC) CCL17; tumor growth factor (TGF)-beta1; alpha-TNF; beta-TNF; epidermal growth factor (EGF); insulin like growth factor-I (IGF-I), angiogenin, oncostatin M, thrombopoietin, VEGF-A; platelet-derived growth factor (PDGF)-bb; leptin. Pituitary gland tissue lysate was extracted in Cell Lysis Buffer provided by the array adding protease inhibitors as recommended. The array membranes were incubated for 30 min at room temperature in blocking buffer. Two hundred (200) micrograms of each tissue lysate sample were then incubated on the membranes overnight at 4 °C on a rocking platform shaker. Following four washes in wash buffer I and three washes in wash buffer II, the membranes were incubated in Biotin-Conjugated Anti-Cytokines for 2 hours at room temperature. After washing, membranes were incubated in horseradish peroxidase (HRP)-Conjugated Streptavidin for 2 hours at room temperature. Washed arrays were finally incubated with Chemi luminescence Detection reagents and images were captured on Uvitec Cambridge Mini HD (Uvitec Ltd.). Signal density of each spot-on membrane was corrected for background intensity and normalized to positive control spots on reference array. In each assay, a negative control was included. A health control was tested (total tissue protein lysate of human adult pituitary gland Leinco), following the same procedures of the cases

*Anti-pituitary antibodies detection*

APA were detected as previous reported (5), by an indirect immunofluorescence method on monkey hypophysis slides (MHY) and monkey hypothalamus slides (MTH) supplied by Biosystem, S.A. (Barcelona 2010). Serum APA and/or AHA bind to the corresponding antigens present on monkey sections. The antigen-antibody complexes are detected by means of a goat anti-human IgG coniugated with fluorescein isothiocyanate (FITC). IgG FITC was adsorbed with monkey serum to remove non-specific fluorescence. Sera of patients were considered positive for APA and/or AHA starting at a dilution rate of 1:8. Samples were considered positive when a diffuse immunofluorescence pattern showing an intracytoplasmic staining was observed in most fields. In each assay, a positive and negative control was included.

*Radiological features*

Tumor volume was calculated with the ellipsoid method, applying the formula 4/3*π*x*y*z/8, where x, y and z represent the diameters of the tumor in the x-, y- and z-axes (6). Pituitary adenoma was defined invasive in cases of invasion of the sellar floor (involving inferiorly the sphenoid sinus and nasopharynx), or of the cavernous sinus laterally, superiorly (infiltering the arachnoid), anteriorly (ethmoid and orbital region) or posteriorly (in the clivus and rarely, in the posterior fossa) (7). Cavernous sinus invasion was defined radiologically as the Knosp grade of 3 or 4; cavernous sinus invasion had to be confirmed intraoperatively and at radiological follow-up.

*Outcome*

Surgery outcome was classified as a partial or radical excision. Partial excision was defined as evidence of a tumor remnant at surgery and/or at MRI imaging 3 months after surgery; radical excision was defined as no evidence of tumor remnant at surgery and at contrasted pituitary MRI performed 3 months after surgery (8), and normalization of hormone hypersecretion in patients with secreting pituitary tumors. Disease outcome was defined according to specific disease guidelines for lactotroph, somatotroph, corticotroph tumors (9–12).

References

1. **Mete O, Osamura RY, Asa SL.** Pituitary gland: introduction. WHO classification of tumours of endocrine organs [M]. *5th ed. Lyon: IARC Press* 2022.

2. **Chiloiro S, Giampietro A, Gessi M, Lauretti L, Mattogno PP, Cerroni L, Carlino A, De Alessandris QG, Olivi A, Rindi G, Pontecorvi A, De Marinis L, Doglietto F, Bianchi A.** CD68+ and CD8+ immune cells are associated with the growth pattern of somatotroph tumors and response to first generation somatostatin analogs. *J Neuroendocrinol* 2023;35(4). doi:10.1111/jne.13263.

3. **Chiloiro S, Moroni R, Giampietro A, Angelini F, Gessi M, Lauretti L, Mattogno PP, Calandrelli R, Tartaglione T, Carlino A, Gaudino S, Olivi A, Rindi G, De Marinis L, Pontecorvi A, Doglietto F, Bianchi A.** The Multibiomarker Acro-TIME Score Predicts fg-SRLs Response: Preliminary Results of a Retrospective Acromegaly Cohort. *J Clin Endocrinol Metab* 2023. doi:10.1210/clinem/dgad673.

4. **Chiloiro S, Bianchi A, Doglietto F, De Waure C, Giampietro A, Fusco A, Iacovazzo D, Tartaglione L, Di Nardo F, Signorelli F, Lauriola L, Anile C, Rindi G, Maira G, Pontecorvi A, De Marinis L.** Radically resected pituitary adenomas: Prognostic role of Ki 67 labeling index in a monocentric retrospective series and literature review. *Pituitary* 2014;17(3):267–276.

5. **Chiloiro S, Angelini F.** An Overview of Diagnosis of Primary Autoimmune Hypophysitis in a Prospective Single-Center Experience. 2016:1–11.

6. **Tirumani SH, Shinagare AB, O’Neill AC, Nishino M, Rosenthal MH, Ramaiya NH.** Accuracy and feasibility of estimated tumour volumetry in primary gastric gastrointestinal stromal tumours: validation using semiautomated technique in 127 patients. *Eur Radiol* 2016;26(1):286–295.

7. **Serioli S, Doglietto F, Fiorindi A, Biroli A, Mattavelli D, Buffoli B, Ferrari M, Cornali C, Rodella L, Maroldi R, Gasparotti R, Nicolai P, Fontanella MM, Poliani PL.** Pituitary Adenomas and Invasiveness from Anatomo-Surgical, Radiological, and Histological Perspectives: A Systematic Literature Review. *Cancers (Basel)* 2019;11(12):1936.

8. **Chiloiro S, Doglietto F, Trapasso B, Iacovazzo D, Giampietro A, Di Nardo F, de Waure C, Lauriola L, Mangiola A, Anile C, Maira G, De Marinis L, Bianchi A.** Typical and Atypical Pituitary Adenomas: A Single-Center Analysis of Outcome and Prognosis. *Neuroendocrinology* 2015;101(2):143–150.

9. **Fleseriu M, Auchus R, Bancos I, Ben-Shlomo A, Bertherat J, Biermasz NR, Boguszewski CL, Bronstein MD, Buchfelder M, Carmichael JD, Casanueva FF, Castinetti F, Chanson P, Findling J, Gadelha M, Geer EB, Giustina A, Grossman A, Gurnell M, Ho K, Ioachimescu AG, Kaiser UB, Karavitaki N, Katznelson L, Kelly DF, Lacroix A, McCormack A, Melmed S, Molitch M, Mortini P, Newell-Price J, Nieman L, Pereira AM, Petersenn S, Pivonello R, Raff H, Reincke M, Salvatori R, Scaroni C, Shimon I, Stratakis CA, Swearingen B, Tabarin A, Takahashi Y, Theodoropoulou M, Tsagarakis S, Valassi E, Varlamov E V, Vila G, Wass J, Webb SM, Zatelli MC, Biller BMK.** Consensus on diagnosis and management of Cushing’s disease: a guideline update. *Lancet Diabetes Endocrinol* 2021;9(12):847–875.

10. **Petersenn S, Fleseriu M, Casanueva FF, Giustina A, Biermasz N, Biller BMK, Bronstein M, Chanson P, Fukuoka H, Gadelha M, Greenman Y, Gurnell M, Ho KKY, Honegger J, Ioachimescu AG, Kaiser UB, Karavitaki N, Katznelson L, Lodish M, Maiter D, Marcus HJ, McCormack A, Molitch M, Muir CA, Neggers S, Pereira AM, Pivonello R, Post K, Raverot G, Salvatori R, Samson SL, Shimon I, Spencer-Segal J, Vila G, Wass J, Melmed S.** Diagnosis and management of prolactin-secreting pituitary adenomas: a Pituitary Society international Consensus Statement. *Nat Rev Endocrinol* 2023;19(12):722–740.

11. **Giustina A, Biermasz N, Casanueva FF, Fleseriu M, Mortini P, Strasburger C, van der Lely AJ, Wass J, Melmed S, Banfi G, Barkan A, Beckers A, Bidlingmaier M, Boguszewski C, Brue T, Buchfelder M, Chanson P, Chiloiro S, Colao A, Coopmans E, Esposito D, Ferone D, Frara S, Gadelha M, Geer EB, Ghigo E, Greenman Y, Gurnell M, Ho K, Ioachimescu A, Johannsson G, Jørgensen JO, Kaiser UB, Karavitaki N, Katznelson L, Lamberts S, Losa M, Luger A, Luque R, Maffei P, Marazuela M, Neggers S, Pereira A, Persani L, Petersenn S, Reincke M, Salvatori R, Samson SN, Schilbach K, Shimon I, Tsagarakis S, Zatelli MC.** Consensus on criteria for acromegaly diagnosis and remission. *Pituitary* 2024;27(1):7–22.

12. **Wass JAH, Reddy R, Karavitaki N.** The postoperative monitoring of nonfunctioning pituitary adenomas. *Nat Rev Endocrinol* 2011;7(7):431–434.
