# Supplementary material for: Cytokines and chemokines modulate the growth of pituitary adenoma/neuroendocrine tumors: preliminary results of a monocenter prospective pilot study
Source: Pituitary. 2025 Mar 10;28(2):37. doi: 10.1007/s11102-025-01505-4 (PMC11893686; doi:10.1007/s11102-025-01505-4)
Supplement: Supplementary file 2 — Supplementary Material 2 [file 11102_2025_1505_MOESM2_ESM.docx]

Supplementary table 1. Descriptive analysis of cytokines and chemokines in samples of analyzed tumors. *: number of case (percentage); §: median (IQR) na: not applicable

|  | All samples | Gonadotroph tumors | Somatotroph tumors | Corticotroph tumors | Lactotroph tumors |
| --- | --- | --- | --- | --- | --- |
| IL-1B  Positive sample *  Quantitative § | 1  62.7 (0) | 0 | 0 | 1  62.7 (na) | 0 |
| IL-2  Positive sample *  Quantitative § | 8  1123 (1210) | 3  1244 (1382) | 3  1458 (1378) | 2  1072 (927) | 0 |
| IL-3  Positive sample *  Quantitative § | 2  143 (393) | 0 | 1  256 (573) | 1  335 (580) | 0 |
| IL-8  Positive sample *  Quantitative § | 6  1150 (2124) | 3  1242 (710) | 1  1430 (na) | 1  410 (na) | 1  1289 (na) |
| MCP1  Positive sample *  Quantitative § | 4  430 (778) | 2  548 (582) | 1  308 (na) | 0 | 1  1030 (na) |
| EGF  Positive sample *  Quantitative § | 3  350 (873) | 1  175 (430) | 0 | 1  428 (na) | 1  1630 (na) |
| GRO  Positive sample *  Quantitative § | 10  1172 (1547) | 4  880 (713) | 2  470 (653) | 2  1170 (1115) | 2  3812 (3505) |
| GRO-a  Positive sample *  Quantitative § | 1  86 (na) | 0 | 0 | 0 | 1  86 (na) |
| Leptin  Positive sample *  Quantitative § | 1  63 (na) | 0 | 0 | 1  63 (na) | 0 |
| RANTES  Positive sample *  Quantitative § | 15  5470 (5695) | 5  3445 (3770) | 5  3725 (2719) | 3  8165 (6149) | 2  11864 (12942) |
| SDF1  Positive sample *  Quantitative § | 16  4274 (942) | 6  4587 (944) | 5  4328 (1083) | 3  3846 (984) | 2  3844 (842) |
| TARC  Positive sample  Quantitative | 3  236 (510) | 2  440 (682) | 1  230 | 0 | 0 |
| MDC  Positive sample *  Quantitative § | 1  100 (na) | 1  100 (na) | 0 | 0 | 0 |
| VEGF  Positive sample *  Quantitative § | 1  77.9 (na) | 1  77.9 (na) | 0 | 0 | 0 |
| PDGF88  Positive sample *  Quantitative § | 1  91 (na) | 0 | 0 | 0 | 1  91 (na) |

Supplementary table 2. Descriptive analysis of circulating anti-pituitary antibodies (APA) and tumor infiltering immune cells, in whole study cohort and according to PitNETs subtypes na: not applicable

|  | All samples | Gonadotroph tumors | Somatotroph tumors | Corticotroph tumors | Lactotroph tumors |
| --- | --- | --- | --- | --- | --- |
| APA, number (percentage) | 4 | 0 | 1 | 2 | 1 |
| CD138+ cells, median (IQR) | 3 (7) | 1 (1) | 7.5 (25) | 1 (1) | 5 (na) |
| CD68+ cells, median (IQR) | 52 (14) | 55 (73) | 47 (17) | 52 (13) | 105 (na) |
| CD4+ cells, median (IQR) | 3 (5) | 1 (2) | 2.5 (5) | 6 (5) | 8 (na) |
| CD8+ cells, median (IQR) | 4 (14) | 3 (1) | 21.5 (42) | 10 (9) | 4 (na) |
| CD3+ cells, median (IQR) | 13 (10) | 6 (1) | 15.5 (37) | 16 (12) | 12 (na) |
| CD20+ cells, median (IQR) | 3 (29) | 3 (7) | 2 (3) | 40 (39) | 30 (na) |
