# Supplementary figures and images for: Cytokines and chemokines modulate the growth of pituitary adenoma/neuroendocrine tumors: preliminary results of a monocenter prospective pilot study
Source: Pituitary. 2025 Mar 10;28(2):37. doi: 10.1007/s11102-025-01505-4 (PMC11893686; doi:10.1007/s11102-025-01505-4)

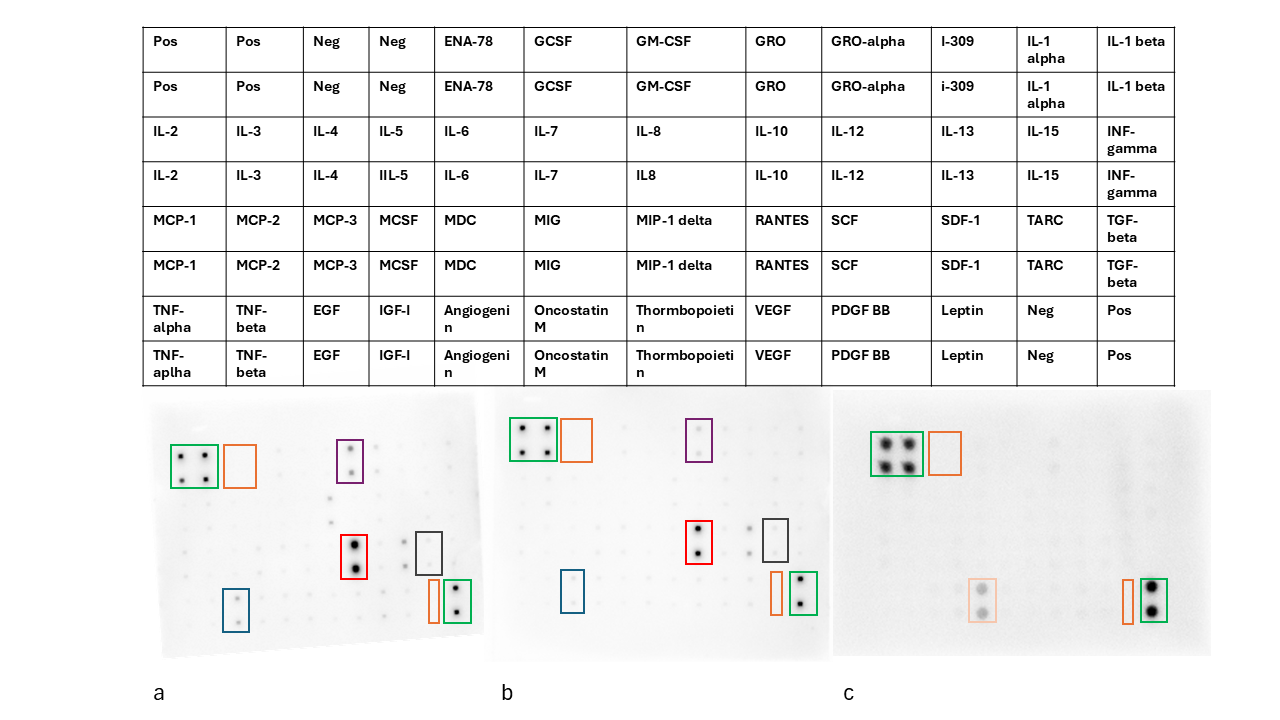

Supplement: Supplementary file 3 — Supplementary Material 3 [file 11102_2025_1505_MOESM3_ESM.tif]

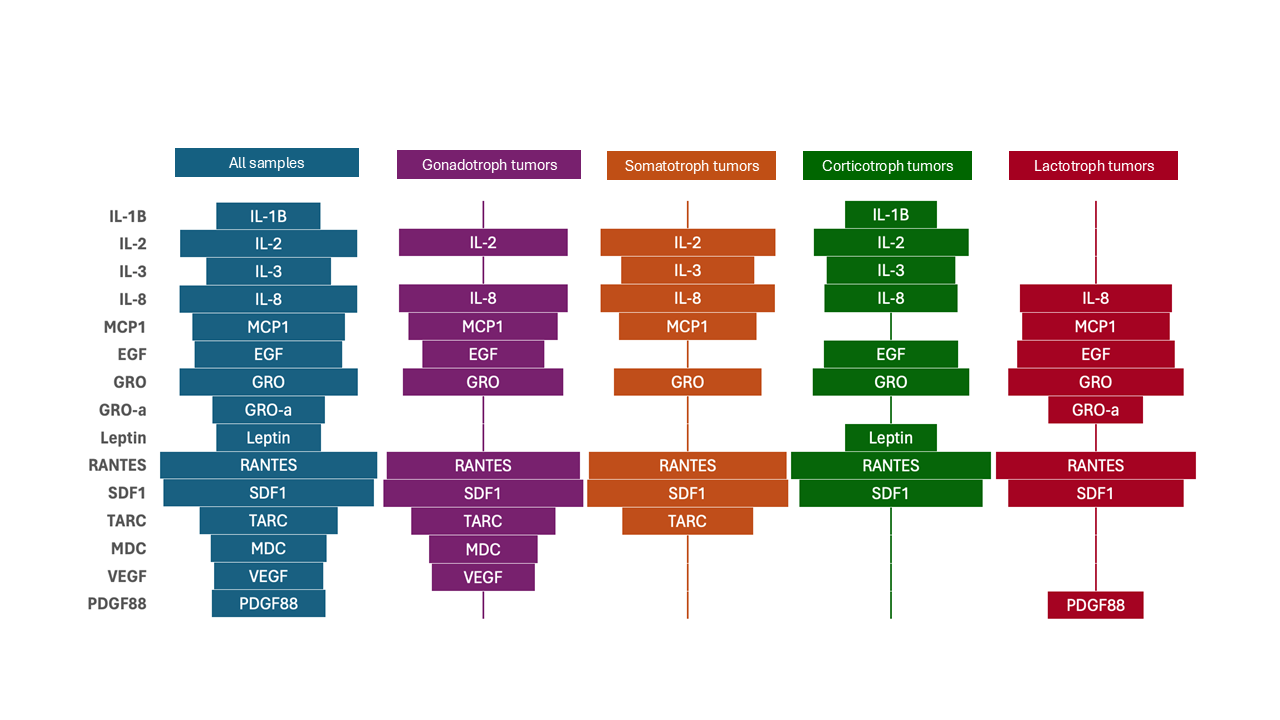

Supplement: Supplementary file 4 — Supplementary Material 4 [file 11102_2025_1505_MOESM4_ESM.tif]
